# Diversity of meningococci associated with invasive meningococcal disease in the Republic of Ireland over a 19 year period, 1996-2015

**DOI:** 10.1371/journal.pone.0228629

**Published:** 2020-02-13

**Authors:** Désirée E. Bennett, Kenneth L. Meyler, Mary T. Cafferkey, Robert J. Cunney

**Affiliations:** 1 Irish Meningitis and Sepsis Reference Laboratory, Children’s Health Ireland, Dublin, Ireland; 2 Department of Microbiology, Royal College of Surgeons in Ireland, Dublin, Ireland; 3 Department of Clinical Microbiology, Children’s Health Ireland, Dublin, Ireland; Universidad Nacional de la Plata, ARGENTINA

## Abstract

This study examined the capsular phenotype and genotype of invasive meningococcal disease (IMD)-associated *Neisseria meningitidis* recovered in the Republic of Ireland (RoI) between 1996 and 2015. This time period encompasses both pre- (when IMD was hyperendemic in the RoI) and post- meningococcal serogroup C conjugate (MCC) vaccine introduction. In total, 1327 isolates representing over one-third of all laboratory-confirmed cases of IMD diagnosed each epidemiological year (EY), were characterised. Serogroups B (menB) and C (menC) predominated throughout, although their relative abundance changed; with an initial increase in the proportion of menC in the late 1990s followed by their dramatic reduction post-MCC vaccine implementation and a concomitant dominance of menB, despite an overall decline in IMD incidence. While the increase in menC was associated with expansion of specific clonal-complexes (cc), cc11 and cc8; the dominance of menB was not. There was considerable variation in menB-associated cc with declines in cc41/44 and cc32, and increases in cc269 and cc461, contributing to a significant increase in the clonal diversity of menB isolates over the study. This increase in diversity was also displayed among the serosubtyping data, with significant declines in proportions of menB isolates expressing p1.4 and p1.15 antigens. These data highlight the changing diversity of IMD-associated meningococci since 1996 in the RoI and emphasise the need for on-going surveillance particularly in view of the recent introduction of a menB vaccine.

## Introduction

*Neisseria meningitidis* (“meningococcus”) is the causative agent of invasive meningococcal disease (IMD) which is responsible for considerable morbidity and mortality throughout the world [1,2]. Based on the immunologic reactivity of their capsular polysaccharides *N*. *meningitidis* can be classified into 12 serogroups, with six (A, B, C, W, Y, and X) being responsible for the majority of IMD cases worldwide, with geographical and temporal variation [1]. In Europe, the Republic of Ireland (RoI) has consistently had one of the highest incidence rates [2,3], increasing from 9.3 per 100,000 total population in epidemiological year (EY; July 1 –June 30) 1997/1998 to 11.6 per 100,000 in EY1999/2000 [4]. During this time disease was predominately associated with serogroups B (menB) and C (menC) [3,4]. However, since the meningococcal serogroup C conjugate (MCC) vaccine was introduced to the routine childhood immunisation schedule in the RoI in October 2000, incidence rates for all forms of IMD (not just serogroup C disease) continuously declined to 1.5 per 100,000 in EY2015/2016 [4], with disease due to menB accounting for >90% of all IMD since 2003, all largely associated with sporadic cases [4]. Therefore, defining isolates by serogroup alone is no longer epidemiologically sufficient and more detailed characterisation is necessary, especially in view of the recent introduction of the multicomponent 4CMenB vaccine into the infant immunisation schedule in the RoI (Dec. 2016). Furthermore, given the diverse nature of menB meningococci in terms of clonal complex distribution [5], it is necessary to accurately define the population of menB strains circulating in the RoI to better assess changes in the epidemiology of meningococcal disease over time, to permit the most efficient allocation of resources and formulation of the most effective disease control and prevention policies.

The objective of this study was to examine the diversity of IMD-associated isolates recovered in the RoI over 19 EYs, since the Irish Meningitis and Sepsis Reference Laboratory (IMSRL) was formally established in October 1996. This timespan includes a period of disease hyperendemicity during the mid-late 1990s (when the levels of disease due to menC were at their highest) up until the end of EY2014/2015 [6]. As part of national surveillance of IMD in RoI, all primary hospital diagnostic laboratories are requested to submit isolates of meningococci recovered from cases of IMD, irrespective of isolation site and clinical presentation, to the IMSRL for confirmation of identity and for epidemiological typing using capsular sero/genogroup, PorB serotype and PorA serosubtype and genotyping (sequence type clonal complex distribution). Between July 1^st^, 1996 and June 30^th^, 2015, a period of 19 EYs, there were 3637 cases of laboratory confirmed IMD reported in the RoI [4].

## Materials and methods

### Bacterial isolates

*N*. *meningitidis* isolates were received as part of routine activity of the IMSRL for performing national surveillance of IMD in the RoI, and were analysed anonymously. Between July 1^st^, 1996 and June 30^th^ 2015, IMSRL received 1327 *N*. *meningitidis* isolates recovered from individual patients diagnosed with laboratory confirmed IMD in the RoI. Of these 1200 were cultured from either blood or cerebrospinal fluid (CSF) and 8 were recovered from synovial fluid. An additional 119 were recovered from non-sterile sites (nose, throat, etc.) of individual patients with IMD that was confirmed by non-culture detection of meningococcal DNA in blood or CSF by PCR.

Four reference *N*. *meningitidis* isolates were included in all analyses. These were the ATCC 13102 serogroup C strain, and a strain of each serogroup A, B and C (M96/255449, H44/76 and C11). The latter were obtained from the Meningococcal Reference Unit, Manchester, U.K.

### Phenotypic characterisation

All 1327 isolates were characterised according to their serogroup and also their PorB serotype and PorA serosubtype as described previously [7–9] using specific panels of antisera (denoted as B 4:NT,p1.4,NT representing serogroup B, serotype 4 with serosubtype VR1,VR2 and VR3 epitopes of NT (non-typeable), p1.4 and NT, respectively).

### Genotypic analysis

To confirm *N*. *meningitidis* species identity and serogroup, all isolates were assayed by PCR amplification for the presence of the conserved meningococcal-specific genes *por*A and *ctr*A, and serogroup-specific genes [10]. Of the 1327 IMD-associated isolates, 1121 (84.5%) were available for molecular characterisation using a variation of the standard 7-locus multi-locus sequence typing (MLST) scheme, multi-locus restriction typing (MLRT), and assigned a restriction type (RT) as described previously [7]. These 1121 isolates comprised of 1018 recovered from either blood or CSF, 8 from synovial fluid specimens and 95 were from non-sterile sites of IMD cases diagnosed by non-culture detection of meningococcal DNA in blood or CSF by PCR. For comparative purposes, MLST was performed on a subsection of these isolates using previously described methods [11–13].Using the RT profile data, sequence type (ST)-clonal complexes (cc) could be inferred to isolates that had not been typed by MLST (Supporting Information).

### Data analysis

Indices of diversity (population richness (R), Simpson’s complement/Gini-Simpson diversity index (Ds) and Shannon entropy (H’) values) were calculated using StatsDirect software, ver. 2.8.0 available from www.statsdirect.com and a ‘Diversity Indices & true diversity’ MSExcel template, ver. 18/12/2012 (author Klaus D. Goepel) downloaded from http://bpmsg.com/?attachment_id=1196. Then from these, values of Pielou’s evenness (Eh = H’/lnR) and clonal diversity (CD = R/number of isolates examined) were calculated [14–16].

Statistical analysis was performed using MS-Excel, version 2010 (Microsoft Corp., Seattle, USA) and Stata, version 14 (StataCorp LP, College Station, Texas, USA). Chi-square analysis was used to test for difference in proportions and analysis of trend examining for overall increase or decrease over the 19 year period was performed using the non-parametric Kendall’s rank correlation coefficient test. Values corresponding to the range, median, 95% confidence interval of median, Kendall’s score and p values are presented for all analysed data parameters. For all statistical analyses p values of < 0.05 were considered significant.

## Results

### Isolates as total of IMD cases/culture positive cases

**Fig 1:**
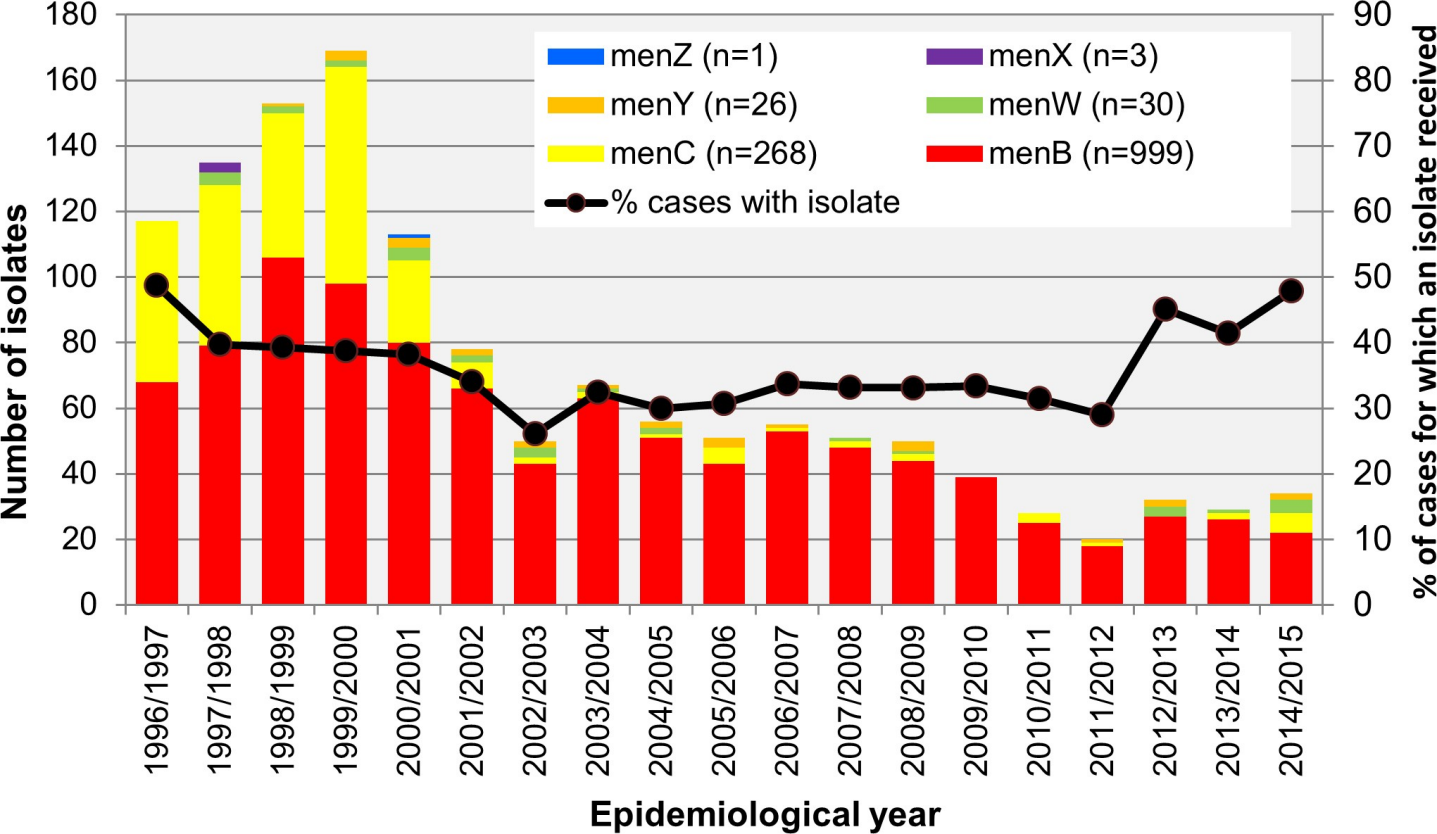
Number and serogroup breakdown of invasive disease-associated *N*. *meningitidis* isolates recovered in Republic of Ireland each epidemiological year (EY) from EY1996/1997 to EY2014/2015.

A total of 1327 *N*. *meningitidis* isolates, each recovered from a separate patient with laboratory confirmed IMD, were received by the IMSRL between 1^st^ July 1996 and 30^th^ June 2015, a period of 19 EYs (Fig 1). It is possible that not all IMD-associated isolates recovered nationally between 1^st^ July and 31^st^ Dec 1996 were received by IMSRL; although, the majority of the isolates (n = 687; 51.9%) were still received during the first five EYs, EY1996/1997 to EY2000/2001 (range 113 to 169 isolates, median 135). There was a decline in the numbers of isolates received each EY since then (Table 1), with a range of 20 to 34 isolates received per EY (median of 29) over the last 5 EY. An isolate was received for 36.5% of all cases over the 19 EYs and the proportion of cases from which a viable isolate was received each EY did not change significantly over the study period (Fig 1 and Table 1). An isolate was received from a median of 39.3% (range 38.2–48.3%) cases during the first 5 EYs and from a median of 43.2% (range 29 to 48.6%) cases over last 5 EYs.

**Table 1:** Descriptive and trend statistics with significance values for prominent phenotypic parameters related to invasive meningococcal disease (IMD)-associated isolates recovered in Republic of Ireland between 1996 and 2015. Data were analysed for trend using the non-parametric Kendall’s rank correlation coefficient test (Stata, version 14; StataCorp LP, College Station, Texas, USA).

Parameter	Descriptive statistics for 19 EYs				KS^a^	p-value
	range	median	95% CI	overall		
**Cases from which a viable *N*. *meningitidis* isolate was phenotyped (received) each EY**						
Number by EY (overall n = 1327)	20–169	51.00	34–113	1327	-129	**0.0000**
Proportion (%) of all IMD cases each EY (overall 1327/3637)	26.04–48.75	33.54	31.46–39.82	36.50%	-25	0.4011
Proportion (%) of menB IMD cases each EY (overall 999/2866)	24.02–58.12	33.54	30.12–39.29	34.90%	-27	0.3630
Proportion (%) of menC IMD cases each EY (overall 268/627)	0–100	40.24	39.06–100	42.70%	6	0.8589
**Proportion of isolates of each serogroup among the isolates phenotyped (received) each EY**						
menB isolates received (overall 999/1327)	57.99–100	86.00	69.28–91.07	75.30%	61	**0.0358**
menC isolates received (overall 268/1327)	0–41.38	6.90	2.99–22.12	20.20%	-67	**0.0208**
menW isolates received (overall 30/1327)	0–11.76	1.96	0–3.54	2.30%	16	0.5933
menY isolates received (overall 26/1327)	0–6.25	1.82	0–5	2.00%	43	0.1345
**Serotype/PorB phenotype**						
**Proportion (%) of all isolates expressing specific PorB antigen/serotype each EY**						
all isolates—PorB antigen/serotype 4	14.71–50	34.48	30.18–39.29	34.6%	-33	0.2629
all isolates—PorB antigen/serotype 15	0–15.63	3.85	2.56–10.71	4.5%	82	**0.0046**
all isolates—PorB antigen/serotype 2a	0–34.91	6.25	3.92–21.57	17.0%	-58	**0.0460**
all isolates—PorB antigen not expressed/non-serotypeable by antisera panel used	17.09–60.78	35.71	28.15–47.06	32%	69	**0.0174**
PorB antigens expressed/serotypes—Richness	5–9	6.00	5–8	9	-91	**0.0011**
PorB—Gini-Simpson index of diversity (Ds)	0.57–0.76	0.72	0.67–0.75	0.742	-41	0.1617
PorB–Shannon entropy (H')	0.99–1.67	1.43	1.24–1.51	1.59	-67	**0.0209**
PorB—Pielou's Evenness (Eh)	0.62–0.89	0.75	0.69–0.82	0.724	29	0.3273
PorB–Clonal diversity (CD) index	0.05–0.3	0.11	0.08–0.15	0.006	116	**0.0001**
**Proportion (%) of menB isolates (n = 999) expressing specific PorB antigen/serotype each EY**						
menB-PorB antigen/serotype 4	22.22–57.35	40.91	33.33–53.16	44.90%	-93	**0.0013**
menB-PorB antigen/serotype 14	0–6.35	0.00	0–3.03	2.10%	-66	**0.0109**
menB-PorB antigen/serotype 15	0–15.38	5.00	3.17–10.42	5.50%	75	**0.0095**
menB-PorB antigen/serotype 21	0–6.25	0.00	0–1.89	1.60%	-61	**0.0147**
menB-PorB antigen not expressed/non-serotypeable by antisera panel used	21.25–62.5	38.89	32.91–48.72	37%	73	**0.0118**
menB–PorB antigens expressed/serotypes—Richness	4–8	6.00	5–7	9	-91	**0.0011**
menB—PorB—Gini-Simpson index of diversity (Ds)	0.56–0.72	0.65	0.61–0.68	0.65	53	0.0689
menB—PorB—Shannon entropy (H')	0.98–1.39	1.24	1.17–1.3	1.34	3	0.9442
menB—PorB—Pielou's Evenness (Eh)	0.6–0.92	0.69	0.64–0.77	0.61	87	**0.0026**
menB—PorB—Clonal diversity (CD) index	0.07–0.28	0.12	0.09–0.15	0.009	103	**0.0004**
**Serosubtype/PorA phenotype**						
**Proportion (%) of all isolates expressing specific PorA VR1,VR2,VR3 antigenic combination/serosubtype each EY**						
all isolates–PorA serosubtype p1.5,P1.2,NT	0–24.85	4	1.96–20.59	13.30%	-48	0.0999
all isolates–PorA serosubtype NT,p1.14,NT	0.74–14.93	8.93	3.92–10.26	6.90%	64	**0.0272**
all isolates–PorA serosubtype NT,p1.15,NT	0–16.34	0.00	0–7.69	6%	-80	**0.0020**
all isolates–PorA serosubtype NT,p1.9,NT	0.85–17.65	7.14	3.27–9.8	5.40%	97	**0.0008**
all isolates–PorA serosubtype p1.19,p1.15,NT	0–17.65	3.57	0–7.84	3.10%	47	0.0934
all isolates–PorA serosubtype NT,p1.10,NT	0–5.19	0.88	0–2.56	2%	-86	**0.0016**
all isolates–PorA serosubtype P1.19,NT,NT	0–7.27	3.57	0–5.13	1.90%	48	0.0863
PorA antigenic combination/serosubtype—richness	10–19	14	13–15	31	-83	**0.0036**
PorA antigenic combination/serosubtype—Gini-Simpson index (Ds)	0.82–0.92	0.86	0.84–0.89	0.87	65	**0.0252**
PorA antigenic combination/serosubtype—Shannon entropy (H')	2.01–2.44	2.2	2.12–2.28	2.52	19	0.5289
PorA antigenic combination/serosubtype—Pielou's Evenness (Eh)	0.77–0.93	0.83	0.78–0.89	0.73	127	**0.0000**
PorA antigenic combination/serosubtype—Clonal diversity (CD) index	0.1–0.5	0.27	0.15–0.38	0.02	134	**0.0000**
**Proportion (%) of menB isolates (n = 999) expressing specific PorA VR1,VR2,VR3 antigenic combination/serosubtype each EY**						
menB—PorA antigenic combination/serosubtype NT,p1.4,NT	13.64–47.06	37.25	27.78–41.86	37%	-89	**0.0021**
menB—PorA antigenic combination/serosubtype NT,p1.15,NT	0–23.58	0.00	0–10.61	7.80%	-86	**0.0009**
menB—PorA antigenic combination/serosubtype NT,p1.9,NT	1.47–16.67	8.75	4.72–11.54	7%	102	**0.0004**
menB—PorA antigenic combination/serosubtype p1.19,p1.15,NT	0–18.75	4	0–9.3	4.10%	47	0.0934
menB—PorA antigenic combination/serosubtype NT,p1.10,NT	0–7.59	1.25	0–2.33	2.10%	-80	**0.0033**
menB—PorA antigenic combination/serosubtype p1.7,p1.16,NT	0–4.55	2.33	1.25–3.8	2.10%	41	0.1595
menB—PorA antigenic combination—richness	9–19	13	11–14	30	-73	**0.0100**
menB—PorA antigenic combination—Gini-Simpson index (Ds)	0.76–0.94	0.82	0.78–0.88	0.83	109	**0.0002**
menB—PorA antigenic combination—Shannon entropy (H')	1.83–2.43	2.05	1.98–2.22	2.4	33	0.2629
menB—PorA antigenic combination—Pielou's Evenness (Eh)	0.71–0.95	0.79	0.75–0.89	0.71	125	**0.0000**
menB—PorA antigenic combination—Clonal diversity (CD) index	0.13–0.59	0.27	0.22–0.37	0.03	117	**0.0000**
**Proportion (%) of menB isolates (n = 999) expressing specific PorA VR2 antigen/serosubtype each EY**						
menB -PorA VR2 p1.4 antigen	13.64–47.06	37.25	27.78–41.86	37.20%	-87	**0.0026**
menB -PorA VR2 p1.15 antigen	0–23.58	10.29	4.65–12.82	12%	-72	**0.0129**
menB -PorA VR2 p1.9 antigen	1.47–16.67	9.09	4.76–12.82	7.60%	97	**0.0008**
menB -PorA VR2 p1.10 antigen	0–7.59	1.25	0–2.33	2.10%	-80	**0.0033**
menB -PorA VR2 antigen not expressed/non-serosubtypeable by panel used	2.5–33.33	12.82	9.09–16.67	12.70%	49	0.0931

^a^KS–Kendall’s Score; CI, Confidence interval; IMD, invasive meningococcal disease; menB, meningococcal serogroup B; menC, meningococcal serogroup C; menW, meningococcal serogroup W; menY, meningococcal serogroup Y.

P value of <0.05 (highlighted in bold text) denotes a significant trend over the 19EY period the direction and extent of which can be inferred by the positive (increasing) or negative (decreasing) Kendall’s score value.

### Serogroup characterisation

Among the 1327 isolates, the serogroup breakdown determined by serology and/or genogrouping PCR was 999 (75%) menB, 268 (20%) menC, 30 (2.3%) menW, 26 (2%) menY, 3 (0.23%) menX and 1 (0.1%) menZ isolate (Fig 1). Only nine isolates were identified as non-groupable by serological methods and these were grouped as menB (n = 8) and menY (n = 1) by PCR. Mirroring the decline in menC IMD, menC isolate numbers declined significantly post 2000, as 233 or 86.9% of all menC isolates were received during the first five EYs (Table 1). MenB accounted for the majority (62.7%) of isolates over the same time period and from then until June 2015, 88.8% of all isolates were menB, or 56.9% of all menB isolates received overall. As expected therefore, the increase in the proportion of menB isolates relative to total number of isolates received over the 19 EY period was significant (Table 1). Since EY 1998/1999, isolates of menW and menY were also received but representing only 2.3% and 2.0% of the total, respectively with non-significant increases in their numbers observed over the 19 EY period (Table 1). Furthermore, there was no change in the proportion of either menB or menC cases from which an isolate was received in relation to the number of IMD cases due to menB or menC, respectively, each EY over the 19 EY period (S1 Table).

### Serogroup, serotype and serosubtype combinations

**Fig 2:**
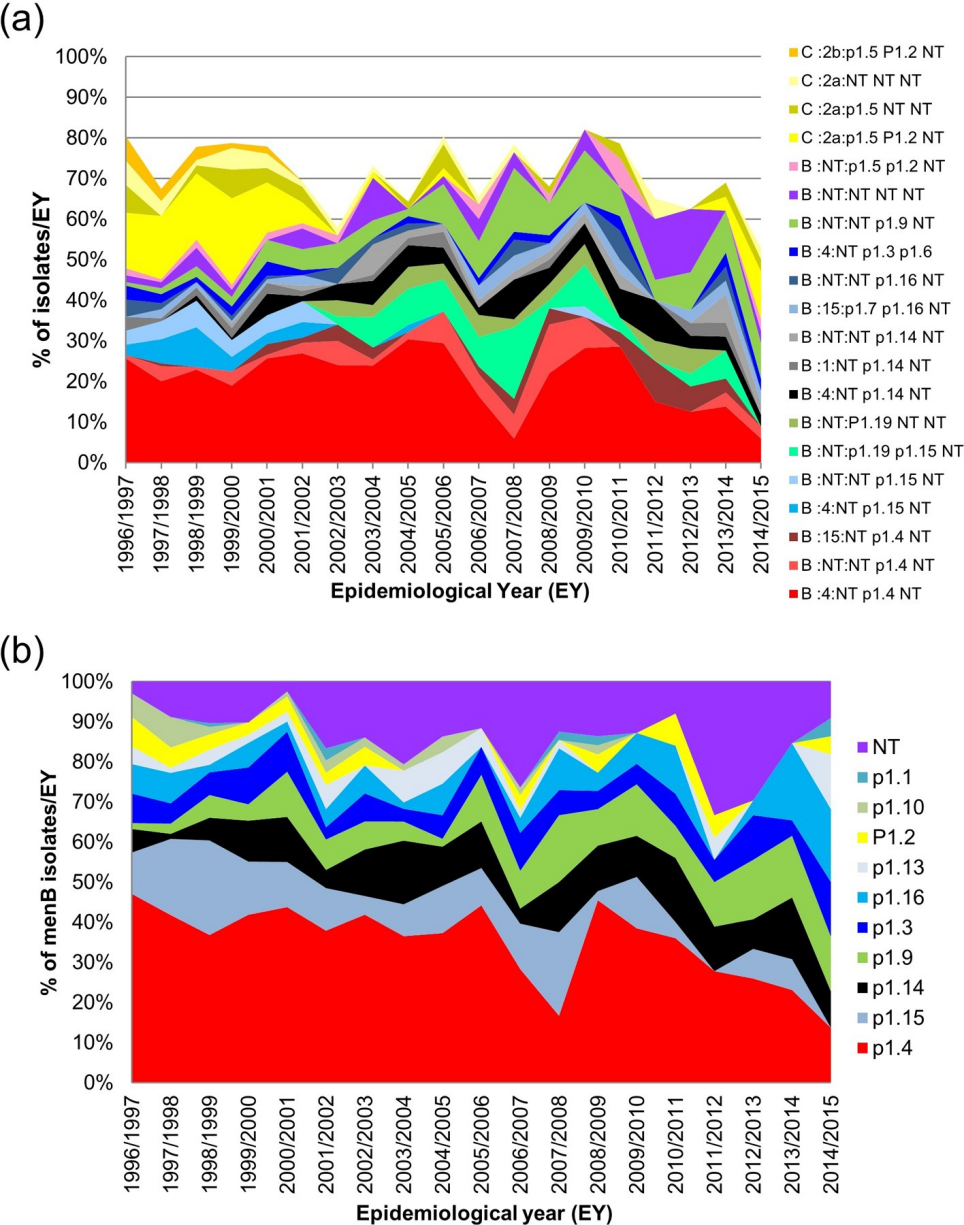
Serological phenotypic profile of invasive disease-associated *N*. *meningitidis* recovered in Republic of Ireland over 19 epidemiological years (EY) between EY1996/1997 and EY2014/2015. (a) Distributions of expressed serogroup, serotype and serosubtype antigenic combinations for the 20 most prevalent combinations each accounting for ≥15 isolates (73% overall) are presented. (b) Distribution of expressed PorA VR2 protein variants presented as the proportion of serogroup B (menB) isolates with each VR2 variant recovered in each EY.

Serological phenotyping identified 144 distinct serogroup, serotype and serosubtype antigenic combinations among the 1327 isolates (Fig 2A); 98 among menB, 27 among menC, 6 among menW, 10 among menY with 2 and 1 among menX and menZ isolates, respectively. The most frequently observed antigenic combination was B 4:NT,p1.4,NT identified in 289 (21.8%) isolates, followed by C 2a:p1.5,p1.2,NT and B NT:NT,p1.9,NT observed in 123 (9.3%) and 68 (5.1%) of isolates, respectively. Overall, 20 distinct antigenic combinations were represented by 15 or more isolates; 16 of which were associated with menB and four with menC (Fig 2A). Among menW and menY isolates, the most common combinations were NT:NT,p1.3,p1.6 and NT:p1.5,NT,NT, representing 36.7% and 23.1% of menW and menY isolates, respectively.

#### Serotype/PorB distribution

A PorB serotype was identified for 67.6% of isolates and the most prevalent serotypes determined are presented in Table 2. The frequency of serotype 4 remained at or above 30% for 16 EYs, dipping in only EY2007/2008 (23.5%), EY2012/2013 (18.8%) and EY2014/2015 (14.7%). In contrast, the prevalence of serotype 2a isolates was above 20% only for the first 5 EYs before dropping to ≤5.5% for 9 of the following 13 EYs resulting in an overall significant decline throughout the study period (Table 1). Although, an increase in serotype 2a isolates to 29.4% (n = 10) was observed in EY2014/2015; this was associated with a combination of menC (n = 6), menW (n = 3) and menB (n = 1) isolates. Other significant changes in porB type observed over the 19 EYs are shown in Table 1. Trend statistics (not included in Table 1) are presented for all serotype, PorA VR2 variant and PorA serosubtype combinations in S1 Table.

**Table 2:** Most prevalent serotypes (PorB antigenic variants) and serosubtypes (PorA antigenic variants) identified among 1327 invasive-disease associated meningococci recovered in Republic of Ireland between EY1996/1997 and EY2014/2015.

Rank order	expressed PorB variant		expressed PorA variant						expressed serosubtype combination	
			VR1		VR2		VR3			
1	4	34.6%	NT	71.0%	p1.4	28.7%	NT	94.3%	NT,p1.4,NT	28.6%
2	NT	32.3%	p1.5	18.2%	NT	17.8%	p1.6	5.7%	p1.5,P1.2,NT	13.3%
3	2a	17.0%	P1.19	6.0%	P1.2	14.7%			NT,NT,NT	9.5%
4	15	4.4%	p1.7	3.5%	p1.15	9.3%			NT,p1.14,NT	6.9%
5	1	4.4%	P1.12	1.3%	p1.14	6.9%			NT,p1.15,NT	6.0%
6	2b	3.0%			p1.3	6.4%			NT,p1.9,NT	5.4%
7	14	2.0%			p1.9	5.9%			NT,p1.3,p1.6	5.4%
8	21	1.7%			p1.16	5.4%			p1.5,NT,NT	4.8%
9	22	0.6%			p1.13	2.5%			NT,p1.16,NT	3.2%
10					p1.10	2.0%			p1.19,p1.15,NT	3.1%
11					p1.1	0.6%			NT,p1.10,NT	2.0%
12									P1.19,NT,NT	1.9%
13									Others (n = 19)^a^	10.0%

^a^The 19 other serosubtype combinations identified in descending order of prevalence were p1.7,p1.16,NT; NT,p1.2,NT; NT,p1.3,NT; P1.19,p1.13,NT; NT,p1.13,NT; P1.7,NT,NT; NT,p1.1,NT; P1.12,P1.16,NT; P1.12,P1.13,NT; NT,NT,p1.6; p1.7,p1.9,NT; p1.12,NT,NT; p1.7,p1.4,NT; p1.7,p1.1,NT; P1.12,P1.9,NT; p1.7,p1.2,NT; P1.7,P1.15,NT; p1.5,p1.16,NT and p1.12,p1.15,NT.

In general, these changes in serotype distribution resulted in an overall non-significant decrease in Gini-Simpson index of diversity (Ds) but a significant decrease in Shannon entropy (H') consistent with a reduction in the number of serotypes identified with an increased dominance of one of two serotypes (either serotype 4 or 2a) and NT in recent years; however the CD index increased significantly throughout the study period (Table 1) indicating that the number of serotypes observed each EY remained similar despite the decreasing number of isolates examined.

Among menB isolates, the frequency of serotype 4 and NT were 44.8% and 37.1%, respectively, overall. Significant changes in the prevalence of serotypes were observed among menB isolates over the 19 EYs with serotype 4, serotype 14 and serotype 21 decreasing and proportions of both serotype 15 and NT increasing significantly (Table 1 and Fig 2B). These changes among the menB population led to increases in the Ds and H' diversity indices and significant increases in Eh and CD values (Table 1). This indicates a reduction in the dominance of specific serotypes and the occurrence of a more evenly distributed population, serotype-wise, over the study period.

#### Serosubtype/PorA distribution

Each of the 1327 isolates was tested using antibodies directed to three PorA epitopes (VR1, VR2 and VR3), and of the 3981 possible PorA epitopes only 1552 (39%) were recognised by the panel of antibodies used at the time of testing. A PorA VR1 was identified for 29% of isolates, a PorA VR2 for 82.2% and a PorA VR3 for only 5.7%. The most prevalent PorA VR1, VR2 and VR3 variants and serosubtype combinations determined are presented in Table 2. Thirty different serosubtype combinations were identified among the 1327 isolates and 9.5% of isolates were non serosubtypeable (NT,NT,NT; Table 2). Over the 19 EYs, the prevalence of isolates with NT,p1.15,NT and NT,p1.10,NT combinations declined significantly with a drop in p1.5,p1.2,NT also observed. In contrast isolates with NT,p1.9,NT increased significantly with increased rates of NT,p1.14,NT, p1.19,p1.15,NT and p1.19,NT,NT also noted (Table 1). Again, these changes in serosubtype frequency among the isolates over time were reflected in increases in all diversity indices (significant for Ds, Eh and CD; Table 1). This indicates the maintenance of the serosubtype richness (number of combinations) each EY despite the decrease in the actual numbers of isolates examined for each EY over the study period.

Among the 999 menB isolates, only 6.6% were NT at all 3 VR epitopes (NT,NT,NT); and the predominant PorA serosubtype combination identified was NT,p1.4,NT (37%), reflecting the predominant p1.4 VR2 variant. This combination was the most prevalent PorA phenotype detected among menB isolates each EY with the exception of EY2007/2008 and EY2014/2015. Changes in PorA serosubtype combinations were very pronounced among the menB isolates, with significant declines observed in rates of isolates with NT,p1.4,NT, NT,p1.15,NT and NT,p1.10,NT combinations with concomitant increases in isolates with NT,p1.9,NT and p1.19,p1.15,NT combinations (Table 1). Even with the high level of non serosubtypeable epitopes, an increase in serosubtype diversity among menB isolates was observed over the 19 EYs demonstrated by increasing Gini-Simpson, Eh and CD indices (Table 1). Again, this reflects the change in the context of the decline in isolate numbers in latter EY to a more even distribution of each of the different serosubtypes away from the predominance of only one or two serosubtypes.

The most common PorA VR1 epitope identified among menB was p1.19 at 8% (81.8% were NT) whereas the most common VR2 epitopes detected were p1.4 (37.2%), p1.15 (12%) and p1.14 (8.8%); 12.7% were NT at VR2 (Fig 2B). Throughout the 19 EYs, there were significant declines in the proportions of menB isolates expressing p1.4, p1.10 and p1.15 epitopes and a significant increase in those expressing the p1.9 epitope (Table 1).

### Genotyping

The 1121 (84.5% of all isolates received) available for genotyping represented 30.8% of all cases of laboratory confirmed IMD diagnosed in the RoI between 1^st^ July 1996 and 30^th^ June 2015 (n = 3637; Table 3). The proportion of cases each EY from which an isolate was analysed ranged from 17.5% to 49.3% (median 33.1%), marginally increasing over the 19 EYs. The 1121 isolates comprised of 854 menB isolates, 211 menC, 28 menW, 25 menY and 3 menX isolates. The overall ratio of the proportion of menB isolates analysed to the proportion of menB cases was calculated at 0.967 for the 19 EY period, ranging from 0.809 in EY 2014/2015 to 1.045 in EY 2009/2010 (median for 19 EYs = 0.966; Table 3). This indicates that for menB, at least at the serogroup level, the analysed isolates were proportionally representative to the proportion of diagnosed cases due to menB. The ratio for menC isolates to cases (excluding both EY2009/2010 and EY2012/2013, when no menC cases were identified) ranged from 0.742 to 3.6 (median 1.2) with an overall value of 1.09. This indicates that a slightly higher proportion of menC isolates were analysed relative to proportion of cases due to menC over the 19 EY period, whereas a disproportionate number of menW and menY isolates compared to menW and menY cases were analysed (Table 3).

**Table 3:** Trend statistics and significance values of genotyping parameters related to invasive meningococcal disease (IMD)-associated isolates recovered in Republic of Ireland over a 19 epidemiological year (EY) period, EY1996/1997 to EY2014/2015. Data were analysed for trend using the non-parametric Kendall’s rank correlation coefficient test (Stata, version 14; StataCorp LP, College Station, Texas, USA).

Genotyping Parameter (n = 1121)	Descriptive statistics for 19 EYs				KS*	p-value
	range	median	95% CI	overall		
% of all cases genotyped (overall 1121/3637)	17.5–49.25	33.11	28.99–35.81	30.82%	53	0.0689
**Representativeness at serogroup level of isolate population genotyped each EY to proportion of cases associated with each serogroup each EY, expressed as a ratio**						
menB isolates genotyped:menB IMD cases (overall 854/1121:2866/3637 = 0.967:1)	0.81–1.05	0.966	0.93–1	0.967	-7	0.8337
menC isolates genotyped:menC IMD cases (overall 211/1121 vs. 627/3637)	0–3.6	1.20	0.99–2.46	1.09	10	0.7527
menW isolates genotyped:menW IMD cases (overall 28/1121 vs. 45/3637)	0–4.19	1.80	0–2.41	2.02	-22	0.4546
menY isolates genotyped:menY IMD cases (overall 25/1121 vs. 37/3637)	0–3.45	1.67	0–2.79	2.19	-6	0.8587
**Proportion (%) of isolates assigned to specific STcc each EY**						
all isolates—cc8	0–16.67	0.00	0–2.34	2.60%	-68	**0.0064**
all isolates—cc11	0–42.86	6.25	4–22.66	16.70%	-65	**0.0252**
all isolates—cc22	0–6.25	3.20	0–3.92	2.90%	-7	0.8307
all isolates—cc23	0–3.13	0.00	0–1.32	0.54%	27	0.2669
all isolates—cc162	0–13.79	0.00	0–1.32	1.10%	40	0.0761
all isolates—cc213	0.96–11.29	3.85	1.96–6.25	3.80%	66	**0.0229**
all isolates—cc269	2.38–37.25	17.31	12.26–25	16.90%	55	0.0589
all isolates—cc461	0–11.76	0.00	0–3.57	1.60%	47	0.0801
all isolates—cc41/44	20.59–51.92	39.47	31.37–45.16	38.50%	11	0.7264
all isolates—assigned STcc- richness (no of STcc)	6–13	10.00	10–11	19	-27	0.3485
all isolates–assigned STcc- Gini-Simpson index (Ds)	0.7–0.86	0.77	0.73–0.82	0.79	53	0.0689
all isolates—assigned STcc- Shannon entropy (H')	1.37–2.04	1.75	1.67–1.97	2.01	45	0.1237
all isolates—assigned STcc- Pielou's Evenness (Eh)	0.64–0.87	0.74	0.73–0.81	0.65	89	**0.0021**
all isolates—assigned STcc- Clonal diversity (CD) index	0.09–0.5	0.20	0.13–0.28	0.02	116	**0.0001**
**Proportion (%) of menB isolates assigned to specific STcc each EY**						
menB—cc32	0–13.89	5.88	4.17–9.46	7.50%	-62	**0.0327**
menB—cc162	0–15.38	0.00	0–1.56	1.40%	40	0.0761
menB—cc 213	1.89–12.07	5.13	2.78–7.69	5%	53	0.0685
menB—cc 269	5–39.58	19.15	17.57–26.56	22%	57	0.0501
menB—cc 461	0–18.18	0.00	0–3.77	2.10%	48	0.0740
menB—cc 41/44	31.82–63.51	50.00	40.74–55.06	50.20%	-79	**0.0064**
menB—assigned STcc—richness (no of STcc)	3–12	8.00	7–10	18	3	0.9431
menB—assigned STcc–Gini-Simpson index (Ds)	0.56–0.85	0.7	0.63–0.75	0.69	77	**0.0078**
menB—assigned STcc—Shannon entropy (H')	0.93–1.84	1.49	1.35–1.69	1.66	59	**0.0424**
menB—assigned STcc—Pielou's Evenness (Eh)	0.59–0.89	0.71	0.68–0.78	0.57	83	**0.0041**
menB—assigned STcc—Clonal diversity (CD) index	0.07–0.44	0.23	0.14–0.28	0.02	91	**0.0016**

*KS–Kendall’s Score; CI, Confidence interval; IMD, invasive meningococcal disease; menB, meningococcal serogroup B; menC, meningococcal serogroup C; menW, meningococcal serogroup W; menY, meningococcal serogroup Y; STcc, sequence type clonal complex (cc).

P value of <0.05 (highlighted in bold text) denotes a significant trend over the 19EY period the direction and extent of which can be inferred by the positive (increasing) or negative (decreasing) Kendall’s score value.

#### Sequence type clonal complex (STcc) distribution

MLST analysis was performed on 383 isolates (28.9% of all isolates received representing 10.5% of all IMD cases) and included 307 menB, 46 menC, 14 menW, 15 menY and one menX isolate. Among these 383 isolates, 189 distinct sequence types (STs) were identified and 158 of these STs were grouped into 21 known international ST clonal complexes (STcc). ST and STcc distribution varied by isolate serogroup (S1 Fig). A total of 163 STs were identified among the 307 menB isolates examined, 136 of these STs, accounting for 267 (87%) of the menB isolates, were assigned to one of 17 STcc while the remaining 27 STs or 40 isolates were not assigned to any STcc by the Neisseria MLST website. Two STcc (cc41/44 and cc269) were responsible for 55.7% of the serogroup B isolates examined. Among the 46 serogroup C isolates examined, 17 distinct STs assigned to 7 different STcc were identified, with 69.6% of isolates belonging to one STcc, cc11.

**Fig 3:**
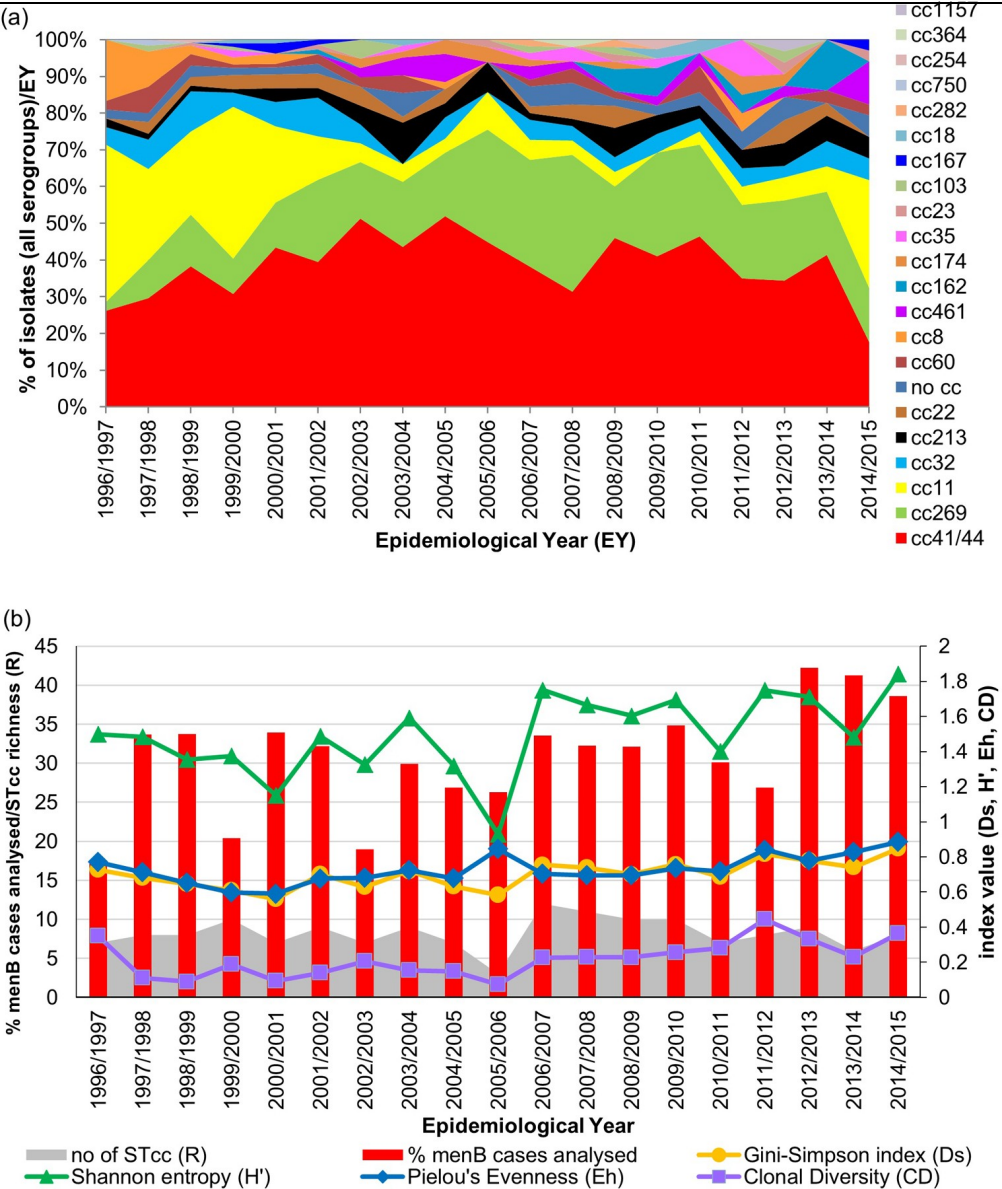
Sequence type clonal complex profile of invasive disease-associated *N*. *meningitidis* isolates recovered in Republic of Ireland over a 19 epidemiological year (EY) period. (a) Distribution of STcc among the 1121 genotyped isolates representing 30.8% of all laboratory confirmed cases of IMD diagnosed in Republic of Ireland between EY1996/1997 and EY2014/2015. (b) Genotypic diversity of IMD-associated menB isolates (n = 854) presented for each EY as the proportion of menB cases diagnosed that were analysed, the number of STcc identified (richness; R), the Gini-Simpson diversity (Ds) index, Shannon entropy (H’), Pielou’s Evenness (Eh) index and clonal diversity (CD) index calculated for each EY. An overall increase in menB isolate diversity as determined by STcc defined by increasing Ds, H’, Eh and CD indices was observed over the study period.

MLRT analysis was performed on all 1121 isolates available for genotyping and included the 373 isolates analysed by MLST and an additional 748 isolates. One hundred and twenty-one distinct restriction types (RTs) were identified which in combination with MLST analysis allowed 97.3% of isolates to be assigned to one of the 21 STcc obtained above. Thirty isolates remained unassigned to a clonal complex despite being designated an ST following MLST analysis (See Supporting Information -S1 Appendix and S1 Fig). The 21 STcc ranged in prevalence from 0.9% for cc364 and cc1157 to 38.6% for cc41/44 (Fig 3A). Three STcc accounted for 72.2% of the isolates; cc41/44 (38.6%), cc269 (17%) and cc11 (16.7%). Isolates of these three STcc were observed in each of the 19 EYs (except no cc11 isolate was identified in EY2009/2010; Fig 3A). Other STccs observed throughout the 19 EYs were cc213 which represented 3.8% of isolates, and cc32 was identified in 17 of the 19 EYs representing 5.9% of isolates.

STcc assignment correlated closely with serogroup: 92.5% of cc11 isolates were menC accounting for 82% of all menC isolates analysed. Meningococci of cc18, cc162, cc213, cc282, cc364, cc461 and cc1157 belonged exclusively to menB as well as 99.3% of cc41/44 isolates. The 25 menY isolates were assigned to just 4 STcc, exclusively for cc23, cc167 and cc174 and 40% of menY isolates belonged to cc174. The 28 menW isolates were assigned to just two STcc with one unassigned to any STcc, 68.8% to cc22 (78.6% of isolates of cc22) and all 5 menW isolates recovered within the last 3 EY were assigned to cc11. In line with isolate serogroup shifts over the 19 EYs, the clonal complex distributions also changed. Significant declines were observed in the proportion of isolates of cc8 and cc11 over the 19 EY period (Table 3) coinciding with the concomitant decline in menC isolates. Little variation was observed over the 19 EYs in the proportion of cc41/44 isolates among the 1121 isolates examined. However, non-significant increases were observed in the proportion of isolates belonging to cc162, cc213, cc269 and cc461. Of these STcc, all of the cc461 isolates (representing only 1.61% of isolates) were recovered since EY2002/2003 and 91.7% of cc162 (accounting for 1.1% of isolates) were observed since EY 2008/2009 (Table 3).

These changing distributions were reflected by an increase in diversity (Ds) from 0.73 to 0.85 (range 0.7–0.86, median 0.77) as well as an increase in H’ statistic from 1.51 to 1.97 (range 1.37 to 2.04, median 1.75), although none were significant. Significant increases were observed though in Eh and CD indices of clonal diversity (Table 3).

The 854 menB isolates analysed grouped into 17 STcc with a predominance of cc41/44 and cc269 accounting for 50.2% and 22.1%, respectively. However, a significant decrease in the proportion of cc41/44 among menB isolates was observed, declining from 50% to 33.3% (absolute range 31.8% to 63.5%; median 50.0%) over the 19 EYs. A decline was also observed over the study period in cc32 menB isolates (range 0 to 13.9%, median 5.88%); whereas increases in the proportions of cc269, cc162 and cc461 menB isolates were noted. Again, these differences in prevalence were reflected in trend statistics (Fig 3B and Table 3), with despite there being a significant decrease in the numbers of menB isolates examined, very significant increases in Ds, Eh and CD were observed as well as an increase in H’ over the 19 EYs. Although, temporary dips in diversity were observed in EY2000/2001, EY2010/2011 and EY2013/2014 but in particular during EY2005/2006 (Fig 3B) due to reduced proportionate numbers of STcc (R and CD) identified. For EY2005/2006, only three STcc were identified among 41 isolates (representing 26.3% of all menB cases diagnosed during that EY), resulting in lower Ds and H’ values but a high Eh as the isolates were almost uniformly distributed between the STcc obtained.

## Discussion

In this report we describe the epidemiology of *N*. *meningitidis* associated with invasive meningococcal disease in the RoI over 19 EYs, between 1^st^ July 1996 and 30^th^ June 2015. This timespan covers the immediate and longer-term impact of the MCC vaccination programme following its introduction in late 2000 and its effects; not only on the menC population but also on the other disease-associated serogroups, including the continuance of menB predominance and also the persistence of menY and menW with transient upsurges [4]. In 1995, prior to the establishment of IMSRL, over half of laboratory confirmed cases of IMD diagnosed in the RoI were due to menB [17] and although the serogroup distribution has changed from year to year since then, menB predominated each EY of the study [3,4]. Nevertheless, the overall case causative serogroup distribution was reflected in the received isolates, throughout the study period, with a predominance of menB isolates received but with varying numbers of isolates of other serogroups also received, proportionate to their case association. In the late 1990s, the number of menC isolates received was at its highest and reflective of the increased incidence of menC cases, similar to that observed in other European countries [1,2]. It was this increased level of menC disease that prompted the introduction of the successful MCC vaccine in the RoI in late 2000 (post its implementation in the UK in 1999), with the consequent decline in the number of notified cases and concomitant reduction in the number of menC isolates received each EY, both of which have remained low. However, the proportion of IMD-associated menC isolates received by IMSRL did increase to 17.6% during EY2014/2015 reflective of the increase in menC cases diagnosed in the RoI in recent EYs (7.1–8.7%; [18]) in line with predicted waning of herd immunity [19,20]. Similarly, the proportions of disease and isolates due to either menW and/or menY also increased within the last 3 EYs of the study, although relative numbers still remain low [18]. Increases in IMD due to menW and menY have also been observed in other European countries due to specific clones [21–27] but this does not appear to be the case in the RoI, at least for menY. For menW, more than 60% of menW isolates received within the last 3 EY of the study were cc11 rather than the traditionally identified cc22 that predominated prior to EY2012/2013; similar to the situation observed in the UK and elsewhere in Europe [2,21,23,27] although without the occurrence of the Hajj pilgrimage-associated MenW:cc11 in the early 2000s [27,28]. In contrast, though in the absence of any obvious intervention measures, the numbers of cases due to and received isolates of menB continued to decline throughout the study period, perhaps attributing inappropriate weight to the increases observed for the other serogroups. Nonetheless, the distribution of serogroups associated with IMD in the RoI [4] is broadly similar to that observed in other European countries for the corresponding time period [2,21].

Substantial PorB and PorA phenotypic diversity was observed among the IMD isolates with a similar wide variety of phenotypes identified in this study as elsewhere [8,29–36] which in general varied annually. A greater number of phenotypes were seen among menB compared to menC isolates although probably due to the greater number of menB isolates and their non-clonal population. The most common PorA phenotype obtained among menB isolates was NT,p1.4,NT and similar to other European countries was the predominant serosubtype among B isolates almost every EY [8,31,35]; although its prevalence declined significantly throughout the 19 EY study period, as also observed in Belgium [36]. However, the persistence of this p1.4 epitope over time is notable given that it is the VR2 region included in the newly introduced 4CMenB (Bexsero®) vaccine [37], though its decline in recent years is worrying. Furthermore, the high proportion of isolates for which no PorA VR serosubtype was identified highlights the low typeability power of the current panel of monoclonal antibodies due to wide variability of the PorA surface protein even within individual VR families (339 VR1 variants and 938 VR2 variants included on http://pubmlst.org/neisseria/PorA accessed 20/06/2019). This underscores the importance of *porA* gene sequencing of the regions encoding the epitopes to deduce the prevailing PorA types circulating among IMD strains as recommended previously [38] which is essential for the design and efficacy estimation of any PorA-based current or future meningococcal vaccine.

In this study, we also assessed the genotypic diversity and relationships of the isolates using STcc assignments inferred following MLRT analysis. The value of MLRT to study the global epidemiology and population structure of several bacterial species has already been established [39–43] and previously applied to meningococcal isolates [7,44,45]. In fact, there was excellent congruence between the STcc assignments following MLRT analysis with the actual STcc as determined by MLST analysis for 373 of the IMD-associated isolates in this study that were analysed by both methods. However, despite MLRT having a lower discriminatory power than that of both standard 7-gene MLST and whole genome sequencing (WGS), its ease of use, short turnaround time and low cost, support its use for initial “in-house” genotypic screening of large collections of isolates. Furthermore, discrimination to the individual ST level by MLST can be too discriminatory to be of global epidemiological value and clustering to STcc level is commonly used to describe meningococcal population structure [5,46,47].

Considerable genetic diversity was observed among our isolates with twenty-one distinct STcc identified, broadly in line with the prevalent disease-associated STcc observed in Europe and worldwide over the same time period [1,5,35]. In our study, three STcc (cc41/44, cc269 and cc11) accounted for almost three quarters of the isolates and these are also the three most prevalent STcc circulating in Europe [5,48–51]. Many of the STcc persisted over the 19 EYs but to varying degrees in each individual EY as observed elsewhere also [36,50,51]. There was an increase in menB STcc heterogeneity during the study contributed to by the decline in prevalence of cc41/44 and cc32 isolates, an increase in cc269 prevalence and the emergence of two STcc (cc461 and cc162) among menB. Isolates of cc41/44, cc269 and cc32 accounted for over 82% of all menB isolates in EY preceding EY2003/2004 and only accounted for 59% of menB isolates in EY2014/2015. A similar decline in these three STcc was also observed in the UK between EY2007/2008 and EY2014/2015 [52]. Although, it is recognised that STcc is not a reliable predictor of antigenic profile in the context of the 4CMenB vaccine coverage estimates [53], it is plausible to suggest that this increase in heterogeneity could indicate a reduction in the likely efficacy of this vaccine now compared to when cc41/44, cc269 and cc32 (which have been demonstrated to have high predicted coverage by the meningococcal antigen typing system- positive bactericidal threshold (MATS-PBT) assay [52,54–56]) accounted for the vast majority of menB IMD [52,56].

As expected following MCC vaccination, menC declined with a corresponding decrease in the prevalence of cc11 and cc8 isolates; 76.5% and 93.1% of cc11 and cc8 isolates, respectively were received prior to EY2001/2002. Reductions in these STcc were also observed in other countries that introduced the MCC vaccine [20,33,36,50,51,57,58]. However, the recovery of cc11 menC and menW in EY2014/2015 is worthy of monitoring, given the extensive association of this STcc with IMD outbreaks [20,57,59,60] and also especially in view of the recent reports of increasing incidence of menW:cc11 in many European countries [23,27,58,61,62].

Over the 19 EYs, the meningococcal population structure associated with IMD in the RoI changed, as despite the lower numbers of isolates received/IMD cases reported each year, an overall increase in genotypic diversity was observed. The predominance of a single STcc has waned in recent years supporting the more sporadic nature of IMD in the RoI [6]. All or at least the vast majority of IMD cases from which an isolate was received over the study period were notified as sporadic with the exception of two isolates from cases that were epidemiologically linked but were recovered in two separate EY [63]. Furthermore, the genotyping data presented here supports the lack of reported epidemiological links between cases from which an isolate was received. Similar increases in genotypic diversity among IMD isolates recovered over similar time frames were also observed in Canada [64], the Netherlands [48] and Belgium [36]. This overall trend of increased diversity for menB masks the transient but marked dips in diversity observed especially during EY2005/2006 and to a lesser extent in EY2000/2001, EY2010/2011 and EY2012/2013. For EY2000/2001, a plausible explanation could be that following vaccination the effect of the removal of menC led to the reduction in diversity due to clonal expansion of the unaffected menB population. Explanations for EY2005/2006, EY2010/2011 and EY2012/2013 are less obvious, although perhaps the “smoking in the workplace” ban introduced in March 2004 which led to reductions in cigarette smoking (both complete cessation and also reduced cigarette consumption among continuing smokers [65–67]; could have impacted on the menB disease strains in EY2005/2006. Smoking and exposure to smoke are known predisposing risk factors for IMD [68]. The transient drops in diversity seen in more recent EY, may possibly be due to the adverse winter weather conditions experienced in both of these EYs; the winter of EY2010/2011 was one of the coldest on record in RoI with several atypical heavy and prolonged snowfalls and the winter of EY2013/2014 was distinguished by severe winter storms with uncharacteristically high rainfall and wind speeds (http://www.met.ie/climate-ireland/major-events.asp). Climate and other external factors have been demonstrated to be associated with trends in IMD in the literature [69,70]. Additionally, seasonal trends in respiratory viral infections (influenzae and RSV) for these two EY were also distinct to preceding years perhaps consistent with reduced levels of population contact [71,72].

It is also reasonable to believe that the demographic change within the population of the RoI could contribute an important role in meningococcal diversity. During the study period, the most dramatic changes in the population of the RoI were observed between the years 2004 and 2008 with year on year increases averaging 2.6% (observed year on year increases averaging 1.4% prior to 2004 and of only 0.5% post 2008; http://www.cso.ie/en/releasesandpublications/er/pme/populationandmigrationestimatesapril2014). A significant contributing factor was an average immigration figure of 114,300 persons/year between 2004 and 2008 (more than twice that observed prior to 2004 and since 2008 of 54,100/year and 56,300/year, respectively). Counterbalancing this was the average yearly emigration figures, increasing throughout the study from 27,500/year for between 1996 and 2004 to 40,200/year between 2004 and 2008 but to 80,000/year between 2008 and 2014. Therefore, even though the population of the RoI continued to increase (overall increase since 1996 of 27.1% by 2014), its composition was very different in 2014 in terms of nationality/country of origin and recent arrival in the RoI compared with 1996, with conceivably the introduction of new/hitherto unseen meningococci to the RoI that contribute to the overall increase in diversity of the IMD-associated isolates being recovered.

This is the first description of the strain characteristics of meningococci associated with IMD in the RoI, examining a comprehensive collection of all IMD isolates received by IMSRL since it was established in 1996. And although an isolate was received from only 36.5% of all laboratory confirmed cases over the 19 EY study, we believe that our data are sufficiently representative to reflect the overall epidemiology of all meningococci associated with IMD in the RoI, irrespective of case ascertainment methodology (I.e. those diagnosed by all non-culture methods). Data comparison with the national computerised infectious disease reporting (CIDR) system, which contains records of all confirmed and suspected cases of IMD notified in the RoI since January 1999, indicated that the vast majority of isolates that are recovered from patients with IMD are actually submitted to the IMSRL [6]. Furthermore, the proportion of cases from which an isolate was recovered was broadly in line with figures described for culture positive cases in the UK for 2009 and 2010 [73]. Therefore, we assume that the results of this study can be applied to the population of IMD strains in the RoI over the past 19 EYs as there is no evidence to suggest that the meningococci associated with culture positive cases may be different to meningococci associated with cases proven by non-culture diagnosis by PCR detection, at least for menB [74].

## Conclusions

The population of invasive meningococci in the RoI is essentially similar to that in other European countries but has certain distinctive elements. The introduction of the MCC vaccine in 2000 virtually eliminated all menC disease and as a result only 10% (n = 27) of all menC isolates received were recovered post EY2001/2002; although, the isolation of 6 (22.2%) of these 27 in EY2014/2015 is very worrying; as are also the recent cases due to cc11 menW meningococci. Of more immediate importance is the increasing trend in genotypic diversity of the isolates, resulting from a reduction in prevalence of menB cc41/44 and associated p1.4 PorA VR2 epitope and also cc32 isolates coupled with a concomitant increase in incidence of cc269 and emergence of cc461 suggesting a reducing value of the newly introduced 4CMenB vaccine. Nevertheless, the data presented here will form a valuable baseline from which the impact of the 4CMenB vaccine in the RoI can measured.

## Supporting information

S1 Appendix Word document detailing concordance between multilocus restriction typing (MLRT) data and multilocus sequence typing (MLST) data. (PDF)

S1 Fig Phylogram of multilocus restriction typing (MLRT) restriction type (RT) profiles demonstrating congruence between MLST clonal complex (STcc) assignment and RT profiles indicating inferred STcc. The phylogram was constructed with the Neighbour-Joining (NJ) algorithm contained in SplitsTree4 (v. 4.10) software. The number of isolates of each RT, the number of isolates of each RT analysed by MLST, the assigned STcc, the inferred STcc and predominant serogroup in each RT are presented. ND- not done; U/A unassigned to cc.
(TIF)

S1 Table Trend statistics and significance values of analysed parameters related to invasive meningococcal disease (IMD)-associated isolates recovered in Republic of Ireland over a 19 epidemiological year (EY) period, EY1996/1997 to EY2014/2015. Data were analysed for trend using the non-parametric Kendall’s rank correlation coefficient test (Stata, version 14; StataCorp LP, College Station, Texas, USA).
(PDF)
