# Supplementary material for: Diversity of meningococci associated with invasive meningococcal disease in the Republic of Ireland over a 19 year period, 1996-2015
Source: PLoS One. 2020 Feb 13;15(2):e0228629. doi: 10.1371/journal.pone.0228629 (PMC7018037; doi:10.1371/journal.pone.0228629)
Supplement: S1 Appendix — (PDF) [file pone.0228629.s001.pdf]

S1 Appendix

Correlation between multilocus restriction typing (MLRT) [1] and multilocus sequence typing (MLST) [2] was established in-house following the analysis of a collection of *N. meningitidis* isolates. The collection comprised of 1922 isolates of known serogroup [3] recovered in Ireland (both IMD-associated isolates and isolates recovered from separate asymptomatic individuals), 40 isolates received as part of External Quality Assurance (EQA) schemes issued from UK NEQAS (National External Quality Assurance scheme) and the four reference strains (ATCC 13102 serogroup C strain, and a strain of each serogroup A, B and C (M96/255449, H44/76 and C11). Each of these 1966 *N. meningitidis* were analysed by MLRT and 247 unique restriction type (RT) profiles were identified [1], and on the basis of sharing 6 of 7 allele digits, a distance matrix was generated using START (v1.0.8) software [1,4]. This matrix was then used to construct a phylogram using the Neighbour-Joining (NJ) algorithm contained in SplitsTree4 (v. 4.10) software [5].

MLST was performed on 725 of these isolates (686 isolates recovered in Ireland [6], 35 EQA isolates & the four reference strains) and their sequence types (STs) and clonal complex (cc) association determined using the Neisseria Multi Locus Sequence Typing website (https://pubmlst.org/neisseria/) [7]. The cc assignment of these isolates was then mapped onto the NJ phylogram constructed from RT profile analysis and significant concordance was observed between RT groupings and STcc assignment with, in general, closely grouped RTs containing isolates of the same STcc (Fig S1). This allowed inference of STcc to isolates of other RTs that had not been typed by MLST. Similar inferences were also made by Dyet et al [8] in their application of MLRT to predict meningococcal ST, albeit using strains of a single cc, cc41/44. Additionally, the topography of the RT-based phylogram was broadly similar to the phylogenetic tree presented by Xu et al. [9] generated based on single nucleotide polymorphisms (SNPs) in 1163 core genes of genome sequences of 23 *N. meningitidis* downloaded from GenBank.

References

1. Bennett DE, Cafferkey MT. Multilocus restriction typing: a tool for Neisseria meningitidis strain discrimination. Journal of medical microbiology. 2003;52: 781–787. doi:10.1099/jmm.0.05225-0

2. Maiden MCJ, Bygraves JA, Feil E, Morelli G, Russell JE, Urwin R, et al. Multilocus sequence typing: a portable approach to the identification of clones within populations of pathogenic microorganisms. Proceedings of the National Academy of Sciences of the United States of America. 1998;95: 3140–5. Available: http://www.pubmedcentral.nih.gov/articlerender.fcgi?artid=19708&tool=pmcentrez&rendertype=abstract

3. Bennett DE, Cafferkey MT. Consecutive use of two multiplex PCR-based assays for simultaneous identification and determination of capsular status of nine common Neisseria meningitidis serogroups associated with invasive disease. Journal of clinical microbiology. 2006;44: 1127–1131. doi:10.1128/JCM.44.3.1127-1131.2006

4. Jolley KA, Feil EJ, Chan MS, Maiden MCJ. Sequence type analysis and recombinational tests (START). Bioinformatics (Oxford, England). 2001;17: 1230–1231. doi:10.1093/bioinformatics/17.12.1230

5. Huson DH, Bryant D. Application of phylogenetic networks in evolutionary studies. Molecular Biology and Evolution. 2006. pp. 254–267. doi:10.1093/molbev/msj030

6. Murphy KM, O'Donnell KA, Higgins AB, O'Neill C, Cafferkey MT. Irish strains of Neisseria meningitidis: characterisation using multilocus sequence typing. British journal of biomedical science. 2003;60: 204–9. Available: http://www.ncbi.nlm.nih.gov/pubmed/14725336

7. Jolley KA, Bray JE, Maiden MCJ. Open-access bacterial population genomics: BIGSdb software, the PubMLST.org website and their applications. Wellcome Open Research. 2018;3: 124. doi:10.12688/wellcomeopenres.14826.1

8. Dyet KH, Simmonds RS, Martin DR. Multilocus Restriction Typing Method to Predict the Sequence Type of Meningococci. Journal of Clinical Microbiology. 2004;42: 1742–1745. doi:10.1128/JCM.42.4.1742-1745.2004

9. Xu Z, Du P, Zhu B, Xu L, Wang H, Gao Y, et al. Phylogenetic study of clonal complex (CC)198 capsule null locus (cnl) genomes: A distinctive group within the species Neisseria meningitidis. Infection, genetics and evolution : journal of molecular epidemiology and evolutionary genetics in infectious diseases. 2015;34: 372–377. doi:10.1016/j.meegid.2015.07.013
