# Supplementary figures and images for: Diversity of meningococci associated with invasive meningococcal disease in the Republic of Ireland over a 19 year period, 1996-2015
Source: PLoS One. 2020 Feb 13;15(2):e0228629. doi: 10.1371/journal.pone.0228629 (PMC7018037; doi:10.1371/journal.pone.0228629)

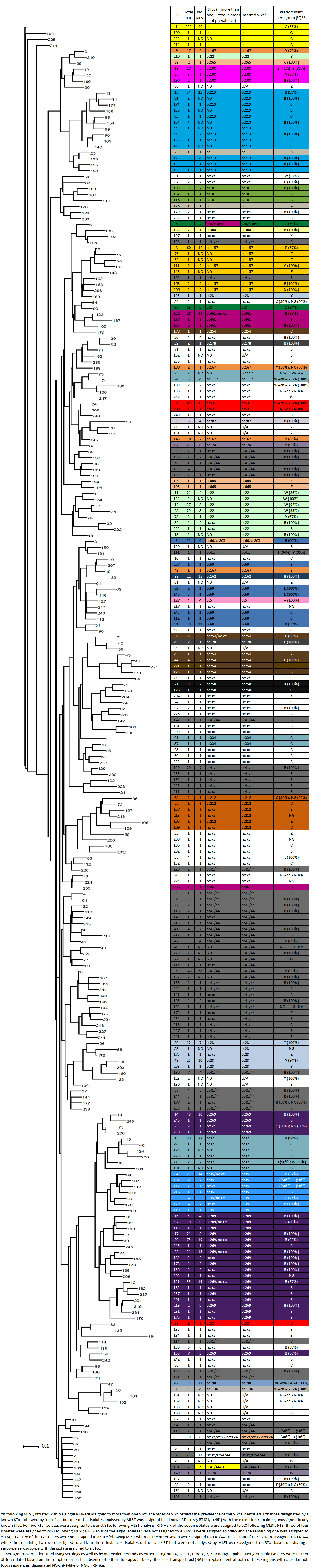

Supplement: S1 Fig — The phylogram was constructed with the Neighbour-Joining (NJ) algorithm contained in SplitsTree4 (v. 4.10) software. The number of isolates of each RT, the number of isolates of each RT analysed by MLST, the assigned STcc, the inferred STcc and predominant serogroup in each RT are presented. ND- not done; U/A unassigned to cc. (TIF) [file pone.0228629.s002.tif]
