# Supplementary material for: Diversity of meningococci associated with invasive meningococcal disease in the Republic of Ireland over a 19 year period, 1996-2015
Source: PLoS One. 2020 Feb 13;15(2):e0228629. doi: 10.1371/journal.pone.0228629 (PMC7018037; doi:10.1371/journal.pone.0228629)
Supplement: S1 Table — Data were analysed for trend using the non-parametric Kendall’s rank correlation coefficient test (Stata, version 14; StataCorp LP, College Station, Texas, USA). (PDF) [file pone.0228629.s003.pdf]

S1 Table. Trend statistics and significance values of analysed parameters related to invasive meningococcal disease (IMD)-associated isolates recovered in Republic of Ireland over a 19 epidemiological year (EY) period, EY1996/1997 to EY2014/2015. Data were analysed for trend using the non-parametric Kendall's rank correlation coefficient test (Stata, version 14; StataCorp LP, College Station, Texas, USA)

Parameter analysed	Descriptive statistics for 19EYs				KS[a]	p-value
	range	median	95% CI	overall		
**Representativeness at serogroup level of isolate population phenotyped (received) each EY to proportion of cases associated with each serogroup each EY, expressed as a ratio**						
menB (overall 999/1327 versus 2866/3637; 0.955:1)	0.81-1.19	0.972	0.93-1	0.955	-43	0.1417
menC (overall 268/1327 versus 627/3637)	0-3.45	1.11	0.97-2.09	1.17	24	0.4207
menW (overall 30/1327 versus 45/3637)	0-3.09	1.67	0-2.51	1.83	-22	0.4546
menY (overall l26/1327 versus 37/3637)	0-3.84	1.54	0-2.94	1.93	-6	0.8587
**Serotype/PorB phenotype (n=1327)**						
**Proportion (%) of isolates expressing specific PorB antigen/serotype each EY**						
all isolates- PorB antigen/sero type 1	0.74-12.5	5.00	3.55-5.97	4.4%	45	0.1233
all isolates- PorB antigen/sero type 14	0-5.97	0.00	0-3.85	2.0%	-53	**0.0415**
all isolates- PorB antigen/sero type 21	0-5.45	0.00	0-2.56	1.7%	-63	**0.0183**
all isolates- PorB antigen/sero type 22	0-3.57	0.00	0-1.49	0.6%	-13	0.6255
all isolates- PorB antigen/sero type 2b	0-10.26	0.00	0-2	3.00%	-68	**0.0109**
**Proportion (%) of menB isolates (n=999) expressing specific PorB antigen/serotype each EY**						
menB - PorB antigen/sero type 1	1.27-13.73	6.12	4-7.35	5.70%	27	0.3624
menB - PorB antigen/sero type 22	0-4	0.00	0-1.59	0.80%	-13	0.6255
menB - PorB antigen/sero type 2a	0-4.55	0.00	0-2.08	1%	-1	1.0000
menB - PorB antigen/sero type 2b	0-5.56	0.00	0-1.96	1.30%	-37	0.1431
**Serosubtype/PorA phenotype (n=1327)**						
**Proportion (%) of all isolates expressing specific PorA VR1,VR2,VR3 antigenic formula/serosubtype each EY**						
all isolates - PorA antigenic formula/serosubtype NT,p1.4,NT	8.82-40	30.97	23.67-34.33	28.60%	-21	0.4841
all isolates - PorA antigenic formula/serosubtype NT,p1.3,p1.6	1.79-8.85	5.23	3.92-7.27	5.40%	28	0.3446
all isolates - PorA antigenic formula/serosubtype p1.5,NT,NT	0-11.76	3.57	1.49-7.69	4.80%	-25	0.3998
all isolates - PorA antigenic formula/serosubtype NT,p1.16,NT	0-7.14	3.92	1.82-5.88	3.20%	25	0.4005
all isolates - PorA antigenic formula/serosubtype p1.7,p1.16,NT	0-3.92	2	0-3.13	1.70%	47	0.1035
all isolates - PorA antigenic formula/serosubtype NT,p1.2,NT	0-4.44	0.00	0-2	1.30%	-30	0.2703
all isolates - PorA antigenic formula/serosubtype NT,p1.3,NT	0-6.25	0.74	0-1.96	1.10%	21	0.4657
all isolates - PorA antigenic formula/serosubtype P1.19,p1.13,NT	0-8.82	0.00	0-1.82	1.10%	14	0.5543
all isolates - PorA antigenic formula/serosubtype NT,p1.13,NT	0-5	0.00	0-1.49	0.98%	-42	0.1191
all isolates - PorA antigenic formula/serosubtype P1.7,NT,NT	0-7.14	0.00	0-1.82	1%	2	0.9688
all isolates - not expressing/non-serosubtypeable for any of the three PorA VR antigens by antisera panel used	0-25	8.82	6-12.73	9.50%	4	0.9164
**Proportion (%) of all isolates expressing specific PorA VR2 antigen/serosubtype each EY**						
all isolates - PorA VR2 p1.4 antigen	8.82-40	30.97	24.85-34.33	28.70%	-21	0.4841
all isolates - PorA VR2 p1.2 antigen	0-26.04	6	1.96-23.53	14.70%	-52	0.0738
all isolates - PorA VR2 p1.15 antigen	0-19.61	7.84	4-10.91	9.30%	-50	0.0863
all isolates - PorA VR2 p1.14 antigen	0.74-14.93	8.93	3.92-10.26	6.90%	64	**0.0272**
all isolates - PorA VR2 p1.3 antigen	3.85-12.5	6.51	5.13-8	6.40%	44	0.1322
all isolates - PorA VR2 p1.9 antigen	0.85-17.65	7.96	3.92-10	5.90%	99	**0.0006**
all isolates - PorA VR2 p1.16 antigen	1.77-17.24	5.45	3.55-8.93	5.40%	65	**0.0252**
all isolates - PorA VR2 p1.13 antigen	0-8.82	1.82	0-5	2.50%	-18	0.5450

all isolates - PorA VR2 p1.10 antigen	0-5.19	0.88	0-2.56	2.0%	-86	**0.0016**
all isolates - PorA VR2 p1.1 antigen	0-2.94	0.00	0-0.88	0.60%	-7	0.8072
all isolates - not expressing a PorA VR2 antigen/non-serotypeable by antisera panel used	11.76-35	18	14.29-20.9	17.80%	37	0.2079
**Proportion (%) of menB isolates (n=999) expressing specific PorA VR1,VR2,VR3 antigenic formula/serosubtype each EY**						
menB - PorA antigenic formula/serosubtype p1.5,P1.2,NT	0-8	2.33	0-3.77	2.50%	-27	0.3493
menB - PorA antigenic formula/serosubtype NT,p1.14,NT	1.27-16	10.26	5.88-11.63	8.90%	44	0.1322
menB - PorA antigenic formula/serosubtype NT,p1.3,p1.6	1.52-13.64	5.66	3.8-7.55	5.70%	15	0.6243
menB - PorA antigenic formula/serosubtype p1.5,NT,NT	0-5.66	1.27	0-3.03	1.60%	4	0.9112
menB - PorA antigenic formula/serosubtype NT,p1.16,NT	0-8	2.53	0-5.88	2.90%	11	0.7237
menB - PorA antigenic formula/serosubtype P1.19,NT,NT	0-7.84	4	0-5.56	2.50%	45	0.1085
menB - PorA antigenic formula/serosubtype NT,p1.3,NT	0-3.92	0.00	0-1.89	1%	6	0.8493
menB - PorA antigenic formula/serosubtype P1.19,p1.13,NT	0-13.64	0.00	0-1.89	1.40%	14	0.5543
menB - PorA antigenic formula/serosubtype NT,p1.13,NT	0-5.56	0.00	0-2.04	1.30%	-46	0.0871
menB - PorA antigenic formula/serosubtype P1.7,NT,NT	0-5.56	0.00	0-3.03	1.20%	-2	0.9688
menB - not expressing/non-serosubtypeable for any of the three PorA VR antigens by antisera panel used	0-22.22	5.06	2.33-11.32	6.60%	36	0.2199
**Proportion (%) of menB isolates (n=999) expressing specific PorA VR2 antigen/serosubtype each EY**						
menB - PorA VR2 p1.2 antigen	0-8	3.75	0-4.65	3.10%	-30	0.2962
menB - PorA VR2 p1.14 antigen	1.27-16	10.26	5.88-11.63	8.90%	44	0.1322
menB - PorA VR2 p1.3 antigen	3.03-13.64	6.25	5.06-9.18	6.70%	16	0.5995
menB - PorA VR2 p1.16 antigen	0-19.23	6.12	2.5-7.84	5.70%	33	0.2623
menB - PorA VR2 p1.13 antigen	0-13.64	2.08	0-5.56	3.30%	-26	0.3734
menB - PorA VR2 p1.1 antigen	0-4.55	0.00	0-0.94	0.60%	12	0.6168
**Genotyping parameter (n=1121)**						
no. of isolates genotyped each EY (n=1121)	20-128	50.00	34-76	1121	-108	**0.0002**
% of menB cases genotyped /EY (B isolates/B cases each EY)	17.09-42.19	32.16	26.86-33.94	29.80%	53	0.0689
% of menC cases genotyped	0-100	40.24	39.06-100	42.70%	6	0.8589
% of menB isolates genotyped among all isolates genotyped for each EY (854/1121)	47.62-100	87.18	69.53-90.38	76.20%	67	**0.0209**
% of menC isolates genotyped among all isolates genotyped for each EY (211/1121)	0-52.38	6.90	3.23-3.23	18.80%	-72	**0.0129**
% of menW isolates genotyped among all isolates genotyped for each EY (28/1121)	0-11.76	1.96	0-3.77	2.50%	18	0.5450
% of menY isolates genotyped among all isolates genotyped for each EY (25/1121)	0-6.25	1.92	0-5	2.20%	31	0.2889
% Culture positive isolates received genotyped	35.9-100	97.44	92.54-100	84.50%	109	**0.0001**
% of menB isolates received that were genotyped (854/999)	29.41-100	96.97	91.14-100	85.40%	111	**0.0000**
% menC isolates received genotyped	0-100	100.00	79.55-100	78.40%	30	0.2151
% menW isolates received genotyped	0-100	100.00	0-100	96.70%	-12	0.6467
% menY isolates received genotyped	0-100	100.00	0-100	92.30%	9	0.7487
**Proportion (%) of isolates assigned to specific STcc each EY**						
all isolates - cc18	0-3.57	0.00	0-0.94	0.45%	0	1.0000
all isolates - cc32	0-10.94	5.13	3.85-6.6	5.90%	-27	0.3624
all isolates - cc35	0-10	0.00	0-1.82	0.80%	19	0.4421
all isolates - cc60	0-7.2	2.38	0-3.45	2.70%	-21	0.4796
all isolates - cc103	0-5.13	0.00	0-1.6	0.71%	-5	0.8644
all isolates - cc167	0-2.94	0.00	0-0	0.54%	-8	0.7291
all isolates - cc174	0-5	0.00	0-2.56	0.89	30	0.2561
all isolates - cc254	0-2.56	0.00	0-0	0.18%	-5	0.7909
all isolates - cc282	0-2	0.00	0-0	0.18%	9	0.5960
all isolates - cc 364	0-1.96	0.00	0-0	0.09%	4	0.7842

all isolates – cc750	0-1.6	0.00	0-0	0.18%	-16	0.1709
all isolates - cc1157	0-3.13	0.00	0-0	0.09%	14	0.2353
all isolates - unassigned to STcc	0-6.25	2.56	0.94-5	2.70%	44	0.1302
**Proportion (%) of menB isolates assigned to specific STcc each EY**						
menB - cc8	0-20	0.00	0-0	1.05%	-26	0.2162
menB - cc11	0-4.55	0.00	0-1.89	1.05%	4	0.9065
menB - cc18	0-4	0.00	0-1.35	0.60%	-2	0.9637
menB - cc 22	0-3.7	0.00	0-1.89	0.80%	10	0.7141
menB - cc35	0-11.11	0.00	0-1.72	0.80%	30	0.1871
menB - cc60	0-8	2.94	0-4.55	2.90%	-30	0.3050
menB - cc103	0-5.88	0.00	0-1.89	0.80%	-4	0.8980
menB - cc254	0-2.56	0.00	0-0	0.12%	8	0.5228
menB - cc 282	0-2.27	0.00	0-0	0.23%	9	0.5960
menB - cc364	0-2.08	0.00	0-0	0.12%	4	0.7842
menB - cc1157	0-3.7	0.00	0-0	0.12%	14	0.2353
menB - unassigned to STcc	0-7.41	3.37	0-5	3%	-4	0.9155

[a]KS – Kendall's Score; CI, Confidence interval; IMD, invasive meningococcal disease; menB, meningococcal serogroup B; menC, meningococcal serogroup C; menW, meningococcal serogroup W; menY, meningococcal serogroup Y; STcc, sequence type clonal complex (cc).
P value of <0.05 (highlighted in bold text) denotes a significant trend over the 19EY period the direction and extent of which can be inferred by the positive (increasing) or negative (decreasing) Kendall's score value.
